# Novel Antioxidant Packaging Films Based on Poly(ε-Caprolactone) and Almond Skin Extract: Development and Effect on the Oxidative Stability of Fried Almonds

**DOI:** 10.3390/antiox9070629

**Published:** 2020-07-17

**Authors:** Arantzazu Valdés García, Nerea Juárez Serrano, Ana Beltrán Sanahuja, María Carmen Garrigós

**Affiliations:** Analytical Chemistry, Nutrition and Food Science Department, University of Alicante, PO Box 99, E-03080 Alicante, Spain; nerea.juarez@ua.es (N.J.S.); ana.beltran@ua.es (A.B.S.); mc.garrigos@ua.es (M.C.G.)

**Keywords:** poly(ε-caprolactone), almond skin, oxidative stability, fried almonds, active packaging, antioxidant activity

## Abstract

Antioxidant films based on poly(ε-caprolactone) (PCL) containing almond skin extract (ASE) were developed for food packaging applications. The effect of ASE incorporation on the morphological, structural, colour, mechanical, thermal, barrier and antioxidant properties of the prepared films were evaluated. The structural, tensile and thermal properties of the films were not altered due to ASE addition. Although no significant differences were observed for the oxygen permeability of samples, some increase in water absorption and water vapour permeability was observed for active films due to the hydrophilic character of ASE phenolic compounds, suggesting the suitability of this novel packaging for fatty foods conservation. ASE conferred antioxidant properties to PCL films as determined by the DPPH radical scavenging activity. The efficiency of the developed films was evaluated by the real packaging application of fried almonds at different ASE contents (0, 3, 6 wt.%) up to 56 days at 40 °C. The evolution of peroxide and p-anisidine values, hexanal content, fatty acid profile and characteristic spectroscopy bands showed that active films improved fried almonds stability. The results suggested the potential of PCL/ASE films as sustainable and antioxidant food packaging systems to offer protection against lipid oxidation in foods.

## 1. Introduction

Lipid oxidation is considered to be the main factor limiting the shelf-life and quality of almonds due to their high content in polyunsaturated fatty acids, mainly linoleic acid [1]. The primary and secondary products such as hydroperoxides, aldehydes and ketones, among others formed during the oxidation process are responsible for the rancid flavour present in the oxidized almonds [2]. The aforementioned degradation is also favoured by cooking processes such as frying in which high temperatures impact directly in the quality of almonds. In addition, the initial linoleic acid content in almonds subjected to frying processes has been reported to be higher than in raw ones, due to oil frying medium migration to the sample, causing a more severe food degradation [3]. To overcome this problem, special attention has been paid in recent years to the use of agri-food by-products as new sources of natural antioxidant agents [4]. This new strategy is aligned with the circular economy action plan to reduce food waste implemented by the European Union since around 1/3 of the total food produced worldwide is estimated to be lost or wasted [5].

Natural antioxidants from food wastes have been incorporated in food packaging, avoiding the direct addition of preservatives into the food and reducing the final price of the packaging materials [6]. The use of agri-food by-products as natural additives for the development of active packaging systems has several advantages; such as availability, recyclability, low-cost, environmentally friendly properties, no toxicity and biodegradability [7]. Several reviews [6,8,9] and research works can be found in the recent literature related to the use of natural antioxidants extracted from food waste or by-products such as orange [10,11], potato [12], olive leaf [13] and mango peels [14] for the development of antioxidant food packaging materials. Among the compounds naturally found in these by-products, antioxidants such as α-tocopherol [15], thymol, carvacrol [16], hydroxytyrosol [17], curcumin [18] and hydrolysed cottonseed proteins [19] have also been incorporated into packaging materials to reduce the problems associated with the lipid oxidation of fatty foods. In addition, oxygen scavengers have been considered as active systems to prevent food oxidation processes. In particular, iron-based [20], organic- [21] and inorganic [22]-based, polymer-based [23] and enzyme-based [24] scavenging systems, among others, have been widely used by the food industry with a residual oxygen reduction to less than 0.1% into the food packaging [25]. However, unlike antioxidants coming from food wastes, these substances are not always in line with the new circular economy concept.

In this context, almond skin (AS) comprises the 4.0–8.0 wt.% of the total shelled almond fresh weight [26] which contains up to 50–75% of the total phenols present in the nut, being rich in hydroxybenzoic acids and aldehydes, phenolic acids, flavan-3-ols, flavonol glycosides, flavonol aglycones, flavanones, flavonone glycosides, flavonone aglycones, isoflavones and lignans [27]. In addition, in a previous work, it was confirmed that AS is a potential natural antioxidant that can reduce the lipid oxidation of food [28]. Then, the incorporation of AS extracts with antioxidant compounds into polymer matrices could be an interesting approach to revalorise AS by-products and to avoid lipid oxidation of fatty foods, such as fried almonds.

Poly(ε-caprolactone) (PCL) is a chemically synthesized polymer which is fully biodegradable and can be processed using conventional plastics machinery [29]. Moreover, it has good properties and is also compatible with other materials [30]. Regarding the use of PCL for active packaging applications, Khalid et al. [31] developed biodegradable antimicrobial packaging films based on PCL, starch and pomegranate rind hybrids showing a strong antimicrobial efficiency at higher concentrations of the active compound. Efficient active films incorporating nisin into PCL and polyhydroxybutyrate (PHB) were also obtained [32] improving ham shelf-life. In recent studies, the direct incorporation of an AS residue as reinforcing agent into a PCL polymer matrix was evaluated, obtaining an improvement in mechanical properties, lower thermal enthalpies and higher crystallinity values [33]. In addition, PCL/AS composites have shown better biodegradability behaviour compared to pure PCL [34]. However, to the best of our knowledge, no studies have been found in the literature focused on the incorporation of almond skin extract (ASE) as natural additive into PCL matrices to develop new antioxidant food packaging systems.

The aim of the present study was the development of effective antioxidant composite films for active packaging by the incorporation of different ASE amounts (0, 3, 6 wt.%) into PCL. The effect of the addition of ASE on the morphological, structural, colour, mechanical, thermal and barrier properties of the films was studied. Moreover, the antioxidant capacity of the active films was evaluated by analyzing 1,1-diphenyl-2-picrylhydrazyl (DPPH) radical scavenging ability. Finally, the efficiency of the developed films was also evaluated by real packaging application of fried almonds up to 56 days at 40 °C in a shelf-life study.

## 2. Materials and Methods

### 2.1. Reagents and Materials

Water (ultrapure grade) and ethanol (HPLC grade) were acquired from Merck (Madrid, Spain). Hexanal (98%), p-anisidine reagent (99%), 2,2-diphenyl-1-picrylhydrazyl (DPPH), sodium chloride, methanol (HPLC grade) and n-hexane (96%, GC grade) were supplied by Sigma-Aldrich (Madrid, Spain). Petroleum ether, sodium methylate, sulphuric acid (98%), acetic acid, potassium iodide, sodium thiosulfate (0.1 M), chloroform, isooctane were of analytical or chromatographic grade and were purchased from Panreac (Barcelona, Spain). Standard compounds such as linoleic (C18:2), oleic (C18:1), palmitic (C16:0), palmitoleic (C16:1), stearic (C18:0) and tridecanoic (C13) acid methyl esters; and 4-methyl-2-pentanone were acquired from Sigma-Aldrich (Madrid, Spain) in the purest available form. A commercial poly(ε-caprolactone) PCL-CAPA 6800 (Mn = 80,000, density = 1.1 g cm^−3^) was supplied in pellets by Perstorp Holding AB (Sweden). Guara almond skins (AS) were kindly supplied by Almendras Llopis (Alicante, Spain).

### 2.2. ASE Preparation

Almond skins from Guara almond cultivar were grounded with a high-speed rotor mill at 12,000 rpm (Ultra Centrifugal Mill ZM 200, RETSCH, Haan, Germany). Particles passing through a 0.5 mm sieve were used and they were dried in a laboratory oven at 40 °C for 24 h to reduce its moisture content. ASE was obtained by microwave-assisted extraction (MAE) by applying an analytical method previously developed [28]. Briefly, 4 g of sample, 60 s, 100 W and 60 mL of 70% (*v/v*) ethanol were used for MAE. The obtained extracts were centrifuged at 4500 rpm for 5 min, filtered through a 0.45 μm filter (PVDF, Teknokroma, Barcelona, Spain), made up to 50 mL and kept at −20 °C until further analysis.

### 2.3. Films Preparation

Two binary systems, containing 3 and 6 wt.% of ASE (PCL3 and PCL6, respectively), were obtained by melt blending in a Haake Polylab QC mixer (ThermoFischer Scientific, Waltham, MA, USA) at 80 °C for 5 min at 100 rpm. An additional sample without ASE was also prepared as control (PCL). Before processing, PCL was left in an oven at 50 °C for 20 h to prevent polymer hydrolysis during processing. ASE was introduced in the mixer once the polymer was in the melt state in order to avoid unnecessary losses.

Films were obtained by compression moulding at 120 °C in a hot-plate hydraulic press (Carver Inc., Model 3850, United States of America). Materials were kept between the plates at atmospheric pressure for 5 min until melting and then they were successively pressed under 2 MPa (1 min), 3.5 MPa (1 min) and finally 5 MPa (5 min). The production of edible films by extrusion, injection, blow-molding and heat-pressing processes are increasingly used [35] due to the combination of efficiency and high productivity provided by these thermal processes. The proposed method avoid also the previous preparation of a PCL solution in an organic solvent such as acetone and the subsequent evaluation of the amount of residual solvent present in the final sample which happens by using other processing techniques such as casting. The average thickness of the obtained films was 200 ± 9 µm measured at five random positions using a 293 MDC-Lite Digimatic Micrometer (Mitutoyo, Japan), after 48 h of conditioning at 50% relative humidity (RH) and 23 °C.

### 2.4. Films Characterization

#### 2.4.1. Scanning Electron Microscopy (SEM)

Morphological characterization of cryo-fractured surfaces of films was performed using a JEOL JSM-840 microscope (Peabody, MA, USA) running at 15 kV. Samples were coated with gold under vacuum using a SCD 004 Balzers sputter coater (Bal Tec. AG, Fürstentum, Lichtenstein) prior to scanning in order to increase their electrical conductivity. Images were registered at a magnification of 1000×.

#### 2.4.2. Colour

Film colour parameters on the CIELAB system a*, b* and L* were determined using a KONICA CM-3600d COLORFLEX-DIFF2 colorimeter, (Hunter Associates Laboratory, Inc., Reston, VA, USA). Measurements were taken at five random positions around the film surface. Total colour differences (ΔE*) induced by the incorporation of the active ASE were calculated with respect to the PCL control film according to the Equation (1):

∆E* = [(∆L*)^2^ + (∆a*)^2^ + (∆b*)^2^]^1/2^ (1)

where ∆L*, ∆a* and ∆b* are the difference in L*, a* and b* values, respectively, between PCL control and each sample (PCL3 and PCL6).

#### 2.4.3. Barrier Properties

Water absorption of films was determined, in triplicate, according to UNE-EN ISO 62:2008 standard [36]. Samples (80 × 10 × 4 mm) were dried in a vacuum oven at 23 °C for 4 h, cooled in a desiccator, and then immediately weighed to the nearest 0.001 g. Then, samples were immersed in distilled water and maintained at 23 °C and 50% RH. Samples were taken out at different times, wiped out properly and then reweighed. Water absorption was calculated according to the Equation (2):

WA = [(W_t_ − W_0_)/ W_t_] × 100 (2)

where W_0_ was the sample weight prior to water adsorption experiment and W_t_ was the final mass at a pre-determined time t.

Water vapour permeability (WVP) was determined according to UNE 53097:2002 standard [37]. Samples of 95 mm diameter were fixed with paraffin on the top of aluminium capsules containing anhydrous calcium chloride, and they were placed in a climate chamber (Dycometal, Barcelona, Spain) at 20 ± 1 °C and 50 ± 2% RH. Capsules were periodically weighed until the steady state was reached. WVP measurements were performed in triplicate.

Oxygen transmission rate (OTR) tests were carried out with an oxygen permeation analyser (8500 model Systech Instruments, Metrotec S.A, Spain). Circular films of 14 cm diameter were clamped in the diffusion chamber at 25 °C. Tests were performed in triplicate by introducing O_2_ (99.9% purity) into the upper half of the diffusion chamber while N_2_ was injected into the lower half, where an oxygen sensor was located. Results were expressed as oxygen transmission rate per film thickness (OTR∙e).

#### 2.4.4. Mechanical Properties

Tensile tests were performed at room temperature using a 3340 Series Single Column System Instron Instrument, LR30K model (Fareham Hants, UK) equipped with a 2 kN load cell. Tests were performed in rectangular probes (100 mm length × 10 mm^2^ cross section) with an initial grip separation of 60 mm and crosshead speed of 25 mm min^−1^. Before testing, all samples were equilibrated for 48 h at 50% RH. Tensile strength, elongation at break and elastic modulus were determined following the ASTM D882-09 standard [38]. Five repetitions were performed for each formulation.

#### 2.4.5. Attenuated Total Reflectance-Fourier Transform Infrared Spectroscopy (ATR-FTIR)

ATR-FTIR spectra were collected by using a Bruker Analitik IFS 66 FTIR spectrometer (Ettlingen, Germany) equipped with a Golden Gate Single Reflection Diamond ATR accessory. Films (1 × 1 mm^2^) were directly placed on the ATR crystal area. Spectra were recorded in the absorbance mode from 4000 to 600 cm^−1^, using 64 scans and 4 cm^−1^ resolution, and corrected against the background spectrum of air. Two spectra replicates were obtained for each sample.

#### 2.4.6. Thermal Analysis

Differential scanning calorimetry (DSC) tests were conducted by using a TA DSC Q-2000 instrument (New Castle, DE, USA) under N_2_ atmosphere (50 mL min^−1^). Films (3 mg) were introduced in aluminium pans (40 µL) and they were submitted to the following thermal program: −80 °C to 160 °C (3 min hold), cooling to −80 °C (3 min hold) and heating to 160 °C, all steps at 10 °C min^−1^. Thermal parameters were determined from the second heating scan. Crystallization and melting temperatures (T_c_ and T_m_) were determined at peak temperatures of the corresponding transitions, while the crystallization and fusion enthalpies (Δ*H*_c_ and Δ*H*_m_) were calculated from the area of the corresponding peaks. The glass transition temperature (T_g_) was also determined for all film samples.

Thermogravimetric analysis (TGA) of ASE and films was performed in a TGA/SDTA 851 Mettler Toledo (Schwarzenbach, Switzerland) thermal analyzer. Then, 4 mg of the samples was heated from 30 to 700 °C at 10 °C min^−1^ under N_2_ atmosphere (50 mL min^−1^). The initial degradation temperature (T_ini_), calculated at 5% of weight loss; and temperature of maximum decomposition rate (T_max_) were determined. DSC and TGA analyses were performed in triplicate.

#### 2.4.7. Antioxidant Activity

The potential antioxidant activity of film samples was evaluated in terms of radical scavenging activity by using the stable radical DPPH (2,2-diphenyl-1-picrylhydrazyl) method, as described by Paneva et al. [39]. Film samples (2.00 ± 0.01 mg) were extracted with 1 mL of methanol at room temperature for 20 min. Then, 10 μL of the methanolic extract were added to 2 mL of freshly prepared DPPH solution (60 µM in methanol). The mixture was shaken vigorously at room temperature and the absorbance of the solution was measured at 517 nm with a Biomate-3 UV–VIS spectrophotometer (Thermospectronic, Mobile, AL, USA). DPPH radical absorbs at 517 nm but, upon reduction by an antioxidant or radical compound, its absorption decreases. The decrease in absorbance was measured at 5 min intervals of incubation in darkness until it was stabilized (20 min). All determinations were performed in triplicate. A second extraction was carried out to evaluate the possible release of antioxidant compounds from the polymer matrix to the solvent. The scavenging ability of the stable radical DPPH was calculated at steady state and expressed as percentage of inhibition (%RSA) using the Equation (3) [40,41].

%RSA = [(A_control_ − A_sample_)/ A_control_] × 100 (3)

where A_control_ and A_sample_ are the absorbances of the blank sample at t = 0 min and the tested sample at t = 20 min, respectively.

### 2.5. Packaging of Fried Almonds

Almonds (100 g) were firstly blanched in 150 mL deionised water at 95 °C for 3 min in order to remove the skins from the kernels by hand. Frying was performed by immersion of almonds in sunflower seed oil at 180 °C for 4 min by using a domestic frying pan [3]. Fried almonds were dried with absorbent paper in a desiccator at room temperature and they were immediately packaged in order to protect them against oxidative reactions.

Fried almonds (50 ± 1 g) were placed in a glass Petri dish (14 cm diameter) and wrapped with the developed active films into a sandwich structure package (almonds were located in the middle of two active films being in contact with both of them). The dish was hermetically closed to avoid oxygen incorporation from the external atmosphere during the shelf-life study. A control sample was also prepared by wrapping fried almonds with the neat PCL film without ASE. Samples were placed at 40 ± 1 °C in an oven (Memmert GmbH, Germany) to accelerate the lipid oxidation process.

### 2.6. Oxidative Stability Study of Packaged Fried Almonds

The accelerated oxidative stability of packaged fried almonds was evaluated at 0, 7, 17, 33 and 56 days of treatment by determining, in triplicate, primary and secondary oxidation products (based on peroxide and p-anisidine values, respectively), hexanal content and fatty acid composition.

#### 2.6.1. Peroxide and p-Anisidine Values

Peroxide and p-anisidine values (PV and AV) were determined according to ISO 3960 standard [42] and IUPAC 2.504 method [43], respectively. Almonds were previously submitted to oil extraction by applying an analytical method previously developed [1].

#### 2.6.2. Hexanal Content

Hexanal content was determined by headspace-solid-phase microextraction (HS-SPME) followed by GC analysis. An Agilent 7820A GC System (Palo Alto, CA, USA) equipped with a FID detector was used. Fried almonds were ground using a domestic electric grinder (Moulinex, Barcelona, Spain). Ground samples (1.00 ± 0.01 g) were placed in a 20 mL vial with a micro-stirring bar and they were mixed with 2 mL of NaCl 2M and 40 μL of the internal standard (4-methyl-2-pentanone, 8 mg kg^−1^). The vial was sealed with an aluminium crimp cap provided with a polytetrafluroethylene/silicone septum [3]. A DVB/CAR/PDMS SPME fibre was used (50/30 µm, StableFlex, 1 cm long) mounted to an SPME manual holder assembly (Supelco, Bellefonte, PA). The sample vial was placed in a water bath at 50 °C and 500 rpm. After 10 min of sample equilibration, the SPME fibre was inserted and exposed to the vial headspace. After 30 min of extraction time, the fibre was immediately desorbed into the GC-FID injection port at 250 °C for 12 min (splitless mode). A SPB-5 column (30 m × 0.32 mm × 0.25 µm; Supelco, Bellefonte, PA) was used which was programmed from 50 °C to 70 °C (hold 1 min) at 5 °C min^−1^ to 200 °C (hold 10 min) at 35 °C min^−1^. Helium was used as the carrier gas (1 mL min^−1^) and the FID temperature was 300 °C. Hexanal was quantified by using a standard calibration curve as described elsewhere [44]. Hexanal stock (30 mg kg^−1^) and working solutions were prepared using distilled water as solvent.

#### 2.6.3. Fatty Acid Profile

Fatty acid profile determination was carried out by methylation of the fatty acids present in oil almond samples as described elsewhere [3]. Analysis of FAMEs was performed by using the GC-FID system previously described for hexanal determination. The GC temperature program used was 120 °C to 245 °C (hold 15 min) at 3 °C min^−1^. An amount of 1 µL was injected in the split mode (1:75). Injector and detector temperatures were 250 and 280 °C, respectively. The identification of FAMEs was performed by comparison with GC retention times of standard compounds. The quantification of FAMEs was performed by using internal standard calibration, using tridecanoic acid methyl ester as internal standard (700 mg kg^−1^).

#### 2.6.4. ATR-FTIR

Structural changes in packaged fried samples were assessed by ATR-FTIR by using experimental conditions previously described in Section 2.4.5.

### 2.7. Statistical Analysis

Statistical analysis of experimental data was performed with SPSS commercial software (Version 15.0, Chicago, IL, USA). A one-way analysis of variance (ANOVA) was carried out. Differences between average values were assessed on the basis of confidence intervals using the Tukey test at a confidence level of 95% (*p* < 0.05).

## 3. Results and Discussion

### 3.1. Films Characterization

#### 3.1.1. SEM Analysis

**Figure 1:**
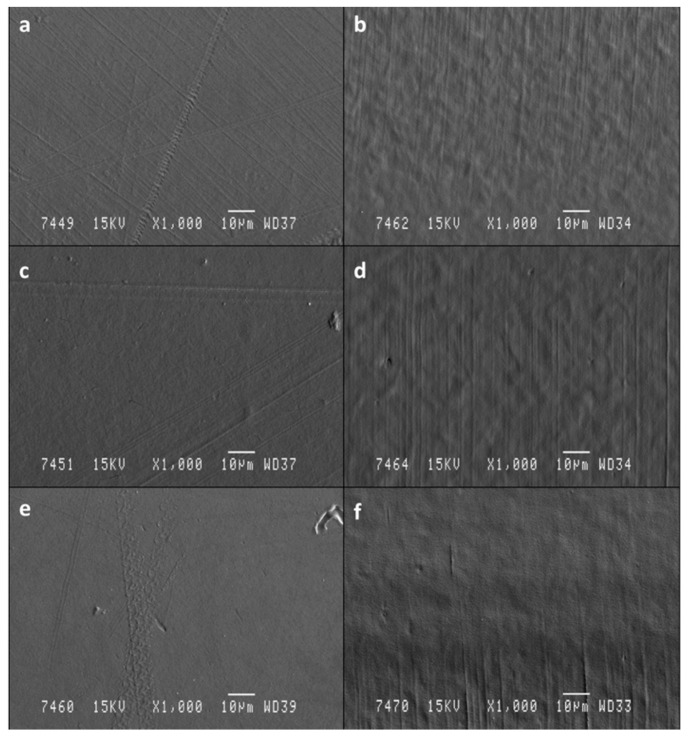
Surface (**a**,**c**,**e**) and cross section (**b**,**d**,**f**) SEM micrographs (1000×) of films ((**a**,**b**): neat PCL; (**c**,**d**): PCL3; (**e**,**f**): PCL6).

SEM micrographs of surface and cross-section of neat PCL and active films (PCL3 and PCL6) are shown in Figure 1. An homogeneous and smooth surface was observed for all films without the presence of pores or cavities. However, some isolated spots (micrographs **c** and **e**) were shown with the addition of ASE to PCL, although no significant structural modifications were observed (micrographs **d** and **f**). This behaviour might be due to the hydrophilicity of polyphenolic compounds present in ASE. Similar microstructural findings were reported for chitosan-based films containing chive root extract [45], agar [46] and carrageenan [47] films incorporated with grapefruit seed extract, and gelatin-based films additivated with curcuma ethanol extract [48]. In conclusion, it was considered that ASE compounds added at the studied concentrations by melting at 80 °C followed by a compression-moulding at 120 °C, as previously described, were dispersed in the polymer matrix uniformly without any significant particles aggregation resulting in homogeneous films with uniform thickness and good appearance.

#### 3.1.2. Colour

**Figure 2:**
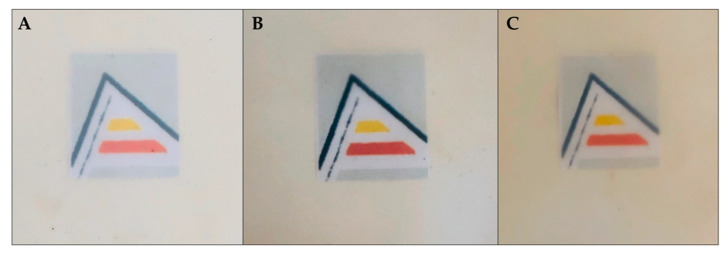
Visual appearance of neat PCL (**A**), PCL3 (**B**) and PCL6 (**C**) films.

The colour of food packaging materials is an important index to be considered in terms of general appearance as it could influence the consumer acceptance [49]. Films containing ASE showed a visual slight yellow colour (Figure 2) in accordance with the significant (*p* < 0.05) decrease in L* (lightness) values and increase in a* and b* values observed with ASE addition (Table 1). Accordingly, the incorporation of ASE to PCL resulted in a remarkable increase in the total colour difference (ΔE*) which was more pronounced with increasing ASE concentration (*p* < 0.05). These differences could be attributed to the natural yellowish colour of ASE. As a result, PCL6 film showed a higher ΔE* value compared to PCL3 formulation. The addition of plant extracts has been reported to modify the original colour of the biopolymers-based films to a certain extent; being the magnitude of this modification determined by the origin of the plant extracts and concentrations used. Phenolic pigments present in plant extracts are likely to contribute to the different colours observed in these biopolymer-based films [50]. Similar results were recently obtained for PCL-based films loaded with sage extract, suggesting that the lower lightness (L*) values obtained with the addition of the extract (from 5 to 20 wt.%) might be helpful to prevent oxidative deterioration in packaged foodstuffs [51]. Moreover, the transparency of the developed films in this work was maintained even for PCL6 film compared to neat PCL, as it can be seen in Figure 2, which is an important issue in food packaging applications.

#### 3.1.3. Barrier Properties

Barrier properties of packaging materials to moisture and oxygen are directly related to their final application and they could affect the food product quality and shelf-life. Water vapour and oxygen are two of the main permeants studied in food packaging applications, as they may transfer from the internal or external environment through the polymer packaging wall, resulting in a continuous change in product quality and shelf-life [52].

The results obtained for OTR, WVP and water absorption properties of the studied films are shown in Table 1. As it can be seen, the addition of ASE to PCL did not significantly affect the OTR values of the obtained films (*p* > 0.05). However, some increase (*p* < 0.05) in WVP and water absorption (after 120 h of immersion) was observed for PCL/ASE films compared to neat PCL. As a result, neat PCL showed a higher hydrophobic behaviour compared to the active films with ASE. Significant decreases in water barrier properties have been reported with the addition of plant extracts to different biopolymer films. Kanmani and Rhim [46] found an increase in WVP values with the addition of grapefruit seed extract in agar-based films which was related to a reduction in intermolecular interactions between the components and the changing pore size of the films. Salevic et al. [51] reported changes in water permeability induced by the addition of sage extract into PCL due to the higher affinity for water of the natural extract in contrast to PCL.

The polyphenolic composition of ASE may determine the final barrier properties of the developed films. In a previous study [28], it was found that ASE extracted from Guara almond cultivar showed high total flavonoids content (1162 μg g^−1^ almond skin) and total phenolic content (119 mg of quercetin equivalent g-1 almond skin), with the isorhamnetin-3-O-rutinoside content being noticeably followed by kaempferol-3-O-rutinoside, quercetin-3-O- rutinoside, naringenin-7-O-glucoside, isorhamnetin-3-O-glucoside, (−)-epicatechin, (+)-catechin and, finally, naringenin contents (Figure S1). Thus, the hydrophilic nature of polyphenols present in ASE may induce an increase in the affinity of active films for water [51,53] by unveiling the availability of hydroxyl groups to bind water molecules [45,54]. Therefore, the developed PCL/ASE films could have potential application as packaging materials for fatty foods according to the increase observed in the solubility in water and WVP of these active films in contrast to neat PCL. Moreover, no significant differences (*p* > 0.05) were observed between samples regarding OTR values. These results are in line with the conclusions supported by other work in which the addition of polyphenols extracted from mango peel into acetylated hemicellulose-nanocellulose-PCL films were suitable for application in packaging of oily or fatty foods [55]. In addition, a similar trend was obtained by Mellinas et al. [15] and Beltrán et al. [17] who reported no significant differences (*p* > 0.05) between PCL-based films with and without the addition of α-tocopherol and hydroxytyrosol, respectively, indicating the suitability of the developed active materials to be used for oxygen-sensitive products.

#### 3.1.4. Mechanical Properties

Film integrity is required in food packaging in order to withstand the stress that may occur during shipping, handling and storage. No significant differences (*p* > 0.05) in mechanical properties were observed with ASE incorporation at 3 and 6 wt.% compared to neat PCL (Table 1). These results are in accordance with the previous characterization performed by SEM and OTR, suggesting that no significant interactions between the polymer matrix and the natural extract compounds in ASE were produced [51]. It is known that materials with thermoplastic behaviour, such as PCL, can be processed into films by the application of different thermal/mechanical dry processing techniques. Therefore, it is essential to study the influence of the selected dry processing on the thermoplastic behaviour of the obtained materials to select adequate processing parameters. In this work, the selected method carried out for the production of PCL-based active films did not significantly affect or modify the final properties of the obtained materials.

On the other hand, variations in mechanical properties of composite films have been directly related to the composition and diversity of chemical compounds present in plant extracts and the biopolymers used. The concentration of the added extract is also an important factor to be considered in order to study possible changes in the physical properties of the composite films [50]. Siripatrawan and Harte [53] prepared chitosan-based films incorporated with green tea extract (GTE) and glycerol as plasticizer reporting no significant differences in mechanical properties of the resulting films compared to the neat polymer at low GTE concentrations (0–5 wt.%). However, tensile strength and elongation at break significantly increased up to 16% and 11% compared to the unfortified controls, respectively, upon the incorporation of GTE from 5 up to 20 wt.%.

#### 3.1.5. ATR-FTIR Analysis

The spectrum of neat PCL showed several characteristic bands around 3450, 2943, 2866, 1724, 1470, 1365, 1180, 1102 and 1045 cm^−1^; in agreement with previous studies [24,56]. Similar bands were found in PCL/ASE films and no significant differences in peaks frequency were observed with the presence of ASE. However, some increase in the absorbance value of the band appearing at 3450 cm^−1^ was observed for PCL6 compared to neat PCL (0.024 ± 0.005 to 0.019 ± 0.006, respectively), which was related to the O-H stretching vibration of hydroxyl groups present in the matrix due to ASE composition [57] (Figure S2). As a result, the availability of OH groups inside the polymer network due to ASE intermingling with PCL was changed [45]. In general, the obtained results suggest that there were no significant chemical bonding between the polymer matrix and ASE, in accordance with previous morphological, mechanical and barrier findings.

#### 3.1.6. Thermal Characterization

**Figure 3:**
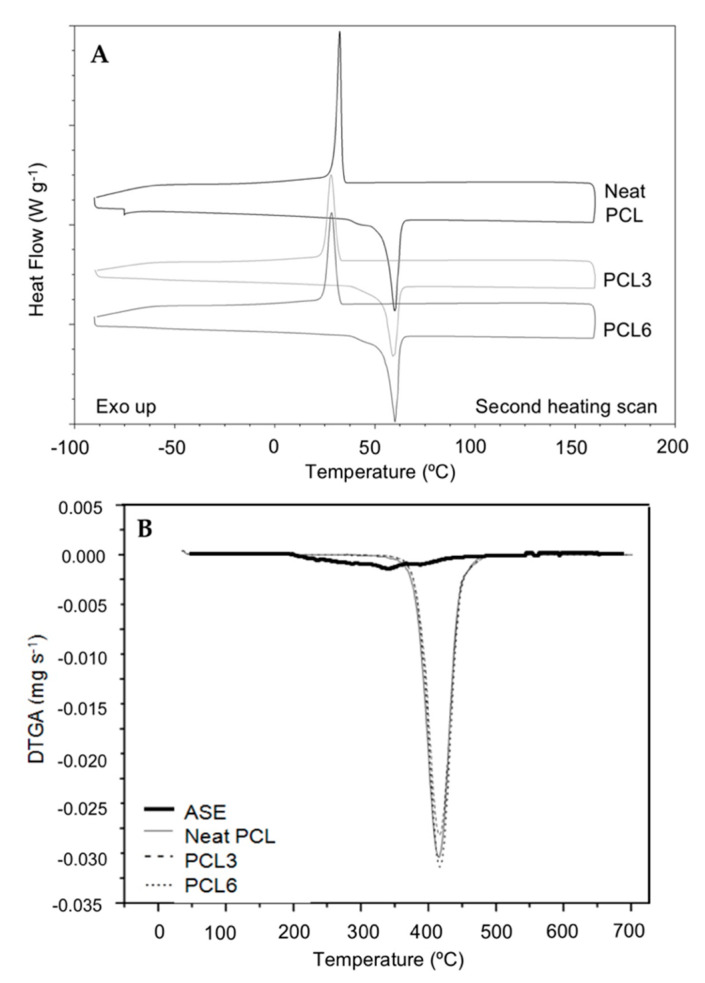
(**A**) DSC curves obtained for neat PCL, PCL3, and PCL6 during the second heating scan in nitrogen atmosphere (10 °C min^−1^); (**B**) DTGA curves for neat PCL, PCL3, PCL6 and ASE in inert atmosphere (10 °C min^−1^).

TGA and DSC tests were carried out to study the influence of ASE addition and the extrusion process on the thermal stability and crystallinity of PCL-based films. All samples presented a characteristic DSC curve showing exothermic crystallization and endothermic melting transitions around 30 and 55 °C, respectively (Figure 3A). Moreover, the T_g_ was obtained around −60 °C [33]. Regarding TGA (Figure 3B), film samples showed one degradation step with initial and maximum temperatures around 385 and 415 °C, respectively. This thermal degradation took place, in inert atmosphere, through the rupture of the polyester chains via ester pyrolysis reaction with the release of CO_2_, H_2_O and carboxylic acids [17]. On the other hand, the DTGA curve obtained for ASE showed one overlapping degradation step with T_ini_ at 139 ± 5 °C and T_f_ at 534 ± 5 °C. ASE T_max_ was obtained at 339 °C ± 4 °C and it was assigned to the degradation of antioxidant compounds with a weight loss of 88% of the total mass. Finally, 3.8 ± 0.2% of residual mass was obtained (Figure S3).

These results are in accordance with the degradation temperatures reported for different antioxidant compounds and extracts. In a previous study, active antioxidant food packaging films were produced by the incorporation of ascorbic acid, ferulic acid, quercetin, and green tea extract into an ethylene vinyl alcohol (EVOH) copolymer. TGA results of films confirmed the presence of degradation peaks at 232 °C for ferulic acid and 249 °C for ascorbic acid which were related to the degradation of antioxidant compounds present in the matrix since the TGA analysis of the pure compounds confirmed the same degradation peaks [58]. A similar thermal stability than that of ASE was reported for curcumin with three degradation steps between 224–239 °C, 439–444 °C and around 570 °C [59]. Recently, Foujdar, Bera and Chopra [60] reported that plant compounds are thermally degraded in three main regions, according to the results obtained for *Punica granatum* peel ethanolic extracts obtained by ultrasonic-assisted extraction. The first region (up to 200 °C) was attributed to moisture loss and evaporation of volatile matters. The second one, up to 400 °C, was mainly due to de-carboxylation and the release of CO_2_ during higher temperatures and, the last one up to hardly 600 °C represented the oxidation of crucial carbon residue.

In this work, no significant differences (*p* > 0.05) were obtained in DSC and TGA parameters among the studied composites containing ASE compared to the neat PCL film (Table 2). These results imply that the addition of ASE did not significantly alter the thermal stability of PCL-based films. Additionally, from Figure 3B, it was concluded that no residual solvent was present in films, since any evaporation of the residual solvent mixture was not observed in the DTGA curves.

#### 3.1.7. Antioxidant Activity

Antioxidant packaging is a very promising alternative for extending food products shelf-life. Enriching films with antioxidants allows nutritional and esthetic quality aspects to be enhanced without affecting the integrity of the food product. The DPPH radical assay has been widely used to test the ability of compounds as free radical scavengers or hydrogen donors in order to evaluate antioxidant activity [53]. Neat PCL film did not show any radical scavenging activity, as expected. On the other hand, films containing 3 and 6 wt.% of ASE presented RSA values of 16 ± 1% and of 20 ± 1%, respectively. No RSA was obtained in any formulation after a second sequential extraction which was related to an efficiently release of the majority of antioxidant compounds from the polymer matrix to the solvent during the first extraction step. As a result, the incorporation of ASE conferred some antioxidant power to PCL/ASE films compared to neat PCL which was attributed to the presence of phenolic compounds in ASE as it has been previously reported in the literature [28]. The free-radical scavenging ability of phenolics is mainly due to their redox properties, which allow them to act as reducing agents, hydrogen donators and singlet oxygen quenchers [53]. Then, the free radical scavenging of ASE could be attributed to the presence of phenolic compounds that can migrate through the polymer matrix to the food product. The obtained results are in accordance with those obtained by Mellinas et al. [15] reporting RSA values around 20% at short initial migration times for PCL-based films added with α-tocopherol by following the DPPH method. Similar results were found for gelatin-based films additivated with curcuma ethanol extract [48], electrospun PCL films with sage natural extract [51], acetylated hemicellulose-nanocellulose-PCL films with mango peel polyphenols [55] and chitosan films containing tea extracts [53], being the obtained RSA values dependent on the type and concentration of the active additive used in the formulations.

The effect of manufacturing conditions is also an important issue to be considered and processing temperatures could play a major role in determining the efficiency of the obtained active films [45,61]. According to DPPH and TGA results, it was concluded that ASE was thermally stable during the whole processing window of PCL/ASE films [59].

### 3.2. Oxidative Stability Study of Packaged Fried Almonds

#### 3.2.1. PV, AV and Hexanal Content

Hydroperoxides are the primary products of lipid oxidation and PV can be used as an oxidative index for the early stages of lipid oxidation. Autoxidation reaction leads to the formation of a broad range of carbonyl compounds, hydrocarbons, furans and other products that contributes to the rancid taste of nuts [62,63]. AV indicates the degree of unsaturation in fat food, being usually used to measure secondary oxidation products formation such as aldehydes. Finally, hexanal content was used as a rancidity indicator since it is a secondary metabolite of lipid oxidation which is formed from the breakdown of linoleic acid peroxides [64].

**Figure 4:**
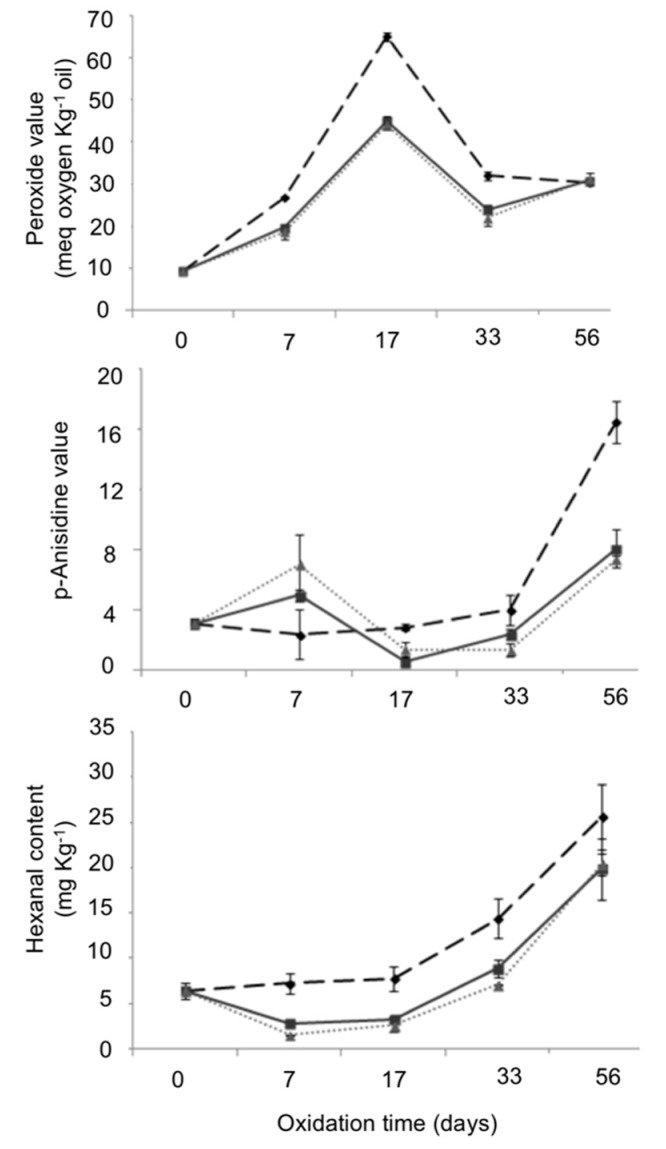
PV, AV and hexanal content for fried almonds packaged with neat PCL (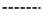), PCL3 (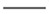) and PCL6 (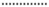) at 0, 7, 17, 33 and 56 days of oxidative treatment at 40 °C. Mean ± SD; n = 3.

Figure 4 shows the evolution of fried almonds packaged with neat PCL and the active PCL/ASE films over the storage time, in terms of PV, AV and hexanal content. The initial PV (day 0) of fried almonds was relatively high (9.4 ± 0.4 meq O_2_ kg^−1^ almond oil) which is indicative of an advanced degree of lipid oxidation as a consequence of almonds processing at high temperatures during frying. Lin et al. reported initial PVs lower than 0.5 meq O_2_ kg^−1^ almond oil for different raw almonds [65]. The peroxide concentration for all samples reached a maximum after 17 days and then it started to decrease up to 56 days. In this case, hydroperoxides may appear only transitorily and they may rapidly decompose into volatile and non-volatile products after 17 days of oxidative treatment. Throughout the experiment, fried almonds packaged in neat PCL showed significant higher PVs compared to the samples stored in active packaging systems. The behaviour shown between 17 and 56 days, with a decrease between 17 and 33 days followed by an increase up to 56 days, may be explained by the fact that peroxides are instable and they can be further degraded to other compounds such as aldehydes and ketones; even while new peroxides are produced [64].

On the other hand, the concentration of aldehydes formed from the peroxides decomposition, measured by AV and hexanal content (Figure 4), significantly increased from day 17 reaching a maximum value at the end of the shelf-life study (56 days). Similar results were obtained by Zajdenwerg et al. [66] when studying the oxidation of Brazil nuts during 21 days of storage at 80 °C, obtaining a maximum PV after 16 days of oxidative treatment and a noticeable increase in AV and hexanal markers from the 12th day. In general, a similar oxidation tendency was observed for neat PCL and PCL/ASE films. Nevertheless, neat PCL showed higher AV and hexanal content at the end of the study (16.5 ± 1.4 and 25.7 ± 2.6 mg hexanal kg^−1^ fried almond, respectively), which can be related to a higher rancidity of fried packaged almonds. Regarding active films, no significant differences (*p* > 0.05) were observed between films containing 3 and 6 wt.% ASE showed AV values of 8.1 ± 1.3 and 7.3 ± 0.2, respectively; and hexanal contents of 19.8 ± 2.4 and 20.4 ± 1.2 mg hexanal kg^−1^ fried almond, respectively. This fact was indicative of a higher extent of oxidation in fried samples packaged into neat PCL compared to samples packaged in active packaging systems which showed some antioxidant effect. These results are in agreement with previous ones obtained for radical-scavenging activity of PCL3 and PCL6 due to the antioxidants present in ASE. Similarly, Pereira de Abreu et al. [62] reported that films containing antioxidants isolated from barley husks were effective in slowing down lipid hydrolysis and primary and secondary lipid oxidation processes of packaged cod fillets during 12 months of frozen storage at −20 °C.

#### 3.2.2. Fatty Acid Profile Determination

Fatty acid composition of fried almonds packaged with neat PCL and the active materials as a function of storage time is shown in Table 3. The most abundant fatty acid at day 0 was oleic acid followed by linoleic acid and smaller amounts of palmitic, stearic and palmitoleic acids. These results are in agreement with previous literature reported for different almond cultivars [3,67].

A general increase in monounsaturated palmitoleic and oleic acids and saturated stearic and palmitic acids content with a parallel decrease in linoleic acid content was observed with increasing oxidation time. This behaviour was more significant for neat PCL compared to the active films (Table 3). Unsaturated fatty acids have different susceptibilities to the abstraction of hydrogens depending on the dissociation energies of the labile C–H bonds. Bonds adjacent to a double bond or to a tertiary carbon atom are weak and easy to break. For the fatty acid series -CH_2_-(CH=CH-CH_2_)n-CH_2_- (*n* = 1–3), the relative oxidation rate increases in a ratio of 1:40:80, respectively, with the oxidation of monounsaturated fatty acids being much less than that of polyunsaturated fatty acids [68]. For this reason, a decrease in linoleic acid as a consequence of oxidation reactions was observed. Since hexanal is a secondary metabolite of lipid oxidation which is formed from the breakdown of linoleic acid hydroperoxides, the higher PV, AV and hexanal contents obtained for samples packaged with neat PCL films could be directly related with the higher linoleic acid oxidation rate shown in Table 3. A decrease in linoleic acid content of 30% was obtained for neat PCL at 56 days of study compared to PCL3 and PCL6 samples which showed lower values (9 and 3%, respectively). These results are in accordance with several reports that linked a higher linoleic acid oxidation rate to a higher formation of peroxides, secondary oxidation products and hexanal content during the study of the oxidative stability of shelled walnuts [64] and frozen cod [62].

#### 3.2.3. ATR-FTIR Analysis

The main bands of fried almonds corresponding to specific functional groups characteristic of almond ingredients (water, fat, protein, and carbohydrates), such as acids, esters, alcohols, and peptides were observed in the ATR-FTIR spectra (3299, 3033, 2923, 2854, 1743, 1627, 1530 and 1045 cm^−1^) [3]. A significant increase was observed (Figure S4) for absorbance values of the bands appearing at approximately 2923 and 2854 cm^−1^ in fried almonds packaged with neat PCL between 7 and 17 days (from 0.360 ± 0.016 to 0.393 ± 0.009 for the band at 2923 cm^−1^, and from 0.231 ± 0.006 to 0.250 ± 0.007 for the band observed at 2854 cm^−1^, respectively). These bands were related to CH_2_ asymmetric and symmetric stretching vibrations. The band at 2923 cm^−1^ was also associated to the saturated fatty acids fraction present in almond samples [67]. So, the observed increase in absorbance for this band could be related to the higher amount of saturated fatty acids observed and thus with a higher oxidation of fried almonds packaged with neat PCL. Finally, no significant differences were observed for fried almonds packaged with PCL3 and PCL6 films during the oxidative treatment which were related to non-significant structural modifications in food samples. As a result, active films delayed the oxidative degradation of fried almonds and maintained their shelf-life for a longer period of time compared to neat PCL. These results are in accordance with PV, AV and hexanal values previously reported. The antioxidant activity of the inferred polyphenols present in ASE could be attributed to assorted mechanisms, including the prevention of radical chain initiation and interaction with free radicals to inhibit lipid oxidation [62].

## 4. Conclusions

Antioxidant films based on PCL and natural antioxidants extracted from almond skin by-products were successfully developed. The incorporation of ASE at 3 and 6 wt.% by melt blending followed by compression moulding at a maximum temperature of 120 °C had a negligible effect on thermal, mechanical and structural characteristics of the resulting films. No deterioration of oxygen barrier properties were observed when comparing the active films with the PCL control, suggesting the suitability of this novel packaging for extending the shelf-life of fatty foods. Results obtained for AV, PV, hexanal content, fatty acid profile and characteristic spectroscopy bands of packaged fried almonds showed that active films effectively delayed their oxidative degradation. In conclusion, antioxidant films based on PCL and ASE at 3 and 6 wt.% have been shown as promising active packaging systems for food preservation, also being an interesting approach to revalorise AS by-products, contributing to the circular economy concept. A scale-up of this study including industrial processing extrusion techniques will be needed in order to obtain commercial products based on these formulations. In addition, further work will be needed in order to evaluate a possible enhancement in antioxidant properties by the addition of higher amounts of ASE and to study the organoleptic behaviour of the packed food in the developed active films to improve the functionality of the obtained formulations.

## Figures and Tables

**Table 1:** The results obtained for colour, barrier and mechanical properties of the studied films.

Property	Neat PCL	PCL3	PCL6
L*	78.0 ± 1.2 ^a^	72.4 ± 0.3 ^b^	61.9 ± 0.4 ^c^
a*	−0.7 ± 0.4 ^a^	3.4 ± 0.2 ^b^	8.5 ± 0.1 ^c^
b*	−1.1 ± 0.2 ^a^	15.9 ± 0.4 ^b^	21.2 ± 0.4 ^c^
ΔE*	-	18.3 ± 0.6 ^a^	29.0 ± 0.6 ^b^
Water absorption (%, 120 h)	0.38 ± 0.09 ^a^	1.05 ± 0.05 ^b^	0.98 ± 0.21 ^b^
OTR (cm^3^ mm m^−2^ day)	95 ± 8 ^a^	109 ± 5 ^a^	115 ± 8 ^a^
WVP × 10^−4^ (kg m Pa^-1^ s^−1^ m^−2^)	1.8 ± 0.1 ^a^	2.4 ± 0.3 ^b^	2.5 ± 0.4 ^b^
Young’s modulus (MPa)	352 ± 20 ^a^	336 ± 20 ^a^	340 ± 15 ^a^
Elongation at break (%)	77 ± 12 ^a^	79 ± 14 ^a^	74 ± 10 ^a^
Tensile strength (MPa)	16 ± 1 ^a^	13 ± 2 ^a^	14 ± 3 ^a^

Colour parameters (L*, a*, b*, ΔE*; mean ± SD; *n* = 5), barrier (mean ± SD; *n* = 3) and mechanical properties (mean ± SD; *n* = 5) of neat PCL and PCL/ASE composite films. Different superscripts within the same row indicate statistically significant different values (*p* < 0.05).

**Table 2:** Thermal properties of neat PCL and PCL/ASE composite films obtained by DSC and TGA.

Thermal property	Neat PCL	PCL3	PCL6
ΔH_c_ (J g^−1^)	59 ± 1 ^a^	62 ± 4 ^a^	59 ± 1 ^a^
T_c_ (°C)	30 ± 1 ^a^	29 ± 1 ^a^	29 ± 1 ^a^
ΔH_m_ (J g^−1^)	59 ± 1 ^a^	58 ± 3 ^a^	57 ± 2 ^a^
T_m_ (°C)	55 ± 1 ^a^	54 ± 1 ^a^	54 ± 2 ^a^
T_g_ (°C)	−61 ± 2 ^a^	−63 ± 2 ^a^	−64 ± 1 ^a^
T_ini_ (°C)	385 ± 2 ^a^	383 ± 3 ^a^	382 ± 3 ^a^
T_max_ (°C)	415 ± 1 ^a^	415 ± 1 ^a^	415 ± 1 ^a^

Thermal properties (mean ± SD; *n* = 3) of neat PCL and PCL/ASE composite films. Different superscripts within the same row indicate statistically significant different values (*p* < 0.05).

**Table 3:** Major fatty acid content of fried almonds (g fatty acid/100 g of almond oil) packaged with neat PCL, PCL3 and PCL6 as a function of storage time at 40 °C.

Time (days)	Fatty Acid	Sample		
		Neat PCL	PCL 3	PCL 6
0	Palmitic	7.91 ± 0.17 ^a^	7.91 ± 0.18 ^a^	7.91 ± 0.18 ^a^
7		8.00 ± 0.65 ^a^	7.75 ± 0.65 ^a^	6.68 ± 0.49 ^b^
17		7.63 ± 2.32 ^a^	6.82 ± 0.14 ^b^	7.50 ± 0.35 ^a,b,c^
33		7.12 ± 0.27 ^a^	6.64 ± 0.03 ^b^	7.40 ± 0.39 ^a,b,c^
56		8.40 ± 0.05 ^a^	6.4 ± 0.24 ^b^	7.31 ± 0.10 ^c^
0	Stearic	7.03 ± 0.56 ^a^	7.03 ± 0.56 ^a^	7.03 ± 0.56 ^a^
7		7.48 ± 0.35 ^a^	9.86 ± 0.35 ^b^	12.61 ± 2.93 ^b,c^
17		11.74 ± 1.52 ^b^	10.93 ± 0.68 ^b^	12.11 ± 0.53 ^b^
33		11.15 ± 0.16 ^b^	9.84 ± 0.47 ^b^	10.08 ± 0.22 ^c^
56		10.77 ± 0.25 ^b^	9.40 ± 0.32 ^b^	9.73 ± 0.70 ^c^
0	Oleic	68.65 ± 0.86 ^a^	68.65 ± 0.86 ^a^	68.65 ± 0.86 ^a^
7		70.56 ± 2.27 ^a^	71.21 ± 2.27 ^a^	67.45 ± 1.08 ^a^
17		68.31 ± 0.88 ^a^	66.92 ± 0.12 ^b^	64.59 ± 1.23 ^b^
33		68.30 ± 0.77 ^a^	68.35 ± 0.51 ^a^	66.28 ± 0.97 ^a,b^
56		69.24 ± 0.06 ^a^	69.08 ± 1.08 ^a^	67.01 ± 0.84 ^a^
0	Linoleic	16.29 ± 0.65 ^a^	16.29 ± 0.65 ^a^	16.29 ± 0.65 ^a^
7		13.86 ± 3.22 ^a,b^	16.37 ± 3.22 ^a^	14.65 ± 0.28 ^b^
17		14.20 ± 0.09 ^b^	15.20 ± 0.44 ^a^	15.68 ± 0.37 ^a^
33		13.28 ± 1.15 ^b^	15.04 ± 0.06 ^a^	16.11 ± 0.43 ^a^
56		11.44 ± 0.08 ^c^	14.85 ± 0.54 ^a^	15.83 ± 0.14 ^a^
0	Palmitoleic	0.114 ± 0.002 ^a^	0.114 ± 0.002 ^a^	0.114 ± 0.002 ^a^
7		0.105 ± 0.010 ^a^	0.132 ± 0.010 ^b^	0.116 ± 0.004 ^a,b^
17		0.117± 0.002 ^a^	0.122 ± 0.001 ^b^	0.120 ± 0.002 ^b^
33		0.145 ± 0.007 ^b^	0.124 ± 0.001 ^b^	0.122 ± 0.004 ^b^
56		0.148 ± 0.004 ^b^	0.120 ± 0.005 ^a,b^	0.122 ± 0.003 ^b^

Mean ± SD; *n* = 3. Different superscripts for each fatty acid within the same column indicate statistically significant different values (*p* < 0.05).

## Supplementary Materials

The following are available online at https://www.mdpi.com/2076-3921/9/7/629/s1, Figure S1: Chemical structure of main polyphenolic composition of ASE, Figure S2: ATR-FTIR analysis of PCL and PCL/ASE composite films, Figure S3: DTGA curve obtained for ASE, Figure S4: ATR-FTIR analysis of fried packaged almonds in PCL and PCL/ASE composite films with storage time.
