# Supplementary material for: Novel Antioxidant Packaging Films Based on Poly(ε-Caprolactone) and Almond Skin Extract: Development and Effect on the Oxidative Stability of Fried Almonds
Source: Antioxidants (Basel). 2020 Jul 17;9(7):629. doi: 10.3390/antiox9070629 (PMC7402149; doi:10.3390/antiox9070629)

Figure S1: Chemical structure of main polyphenolic composition of ASE,

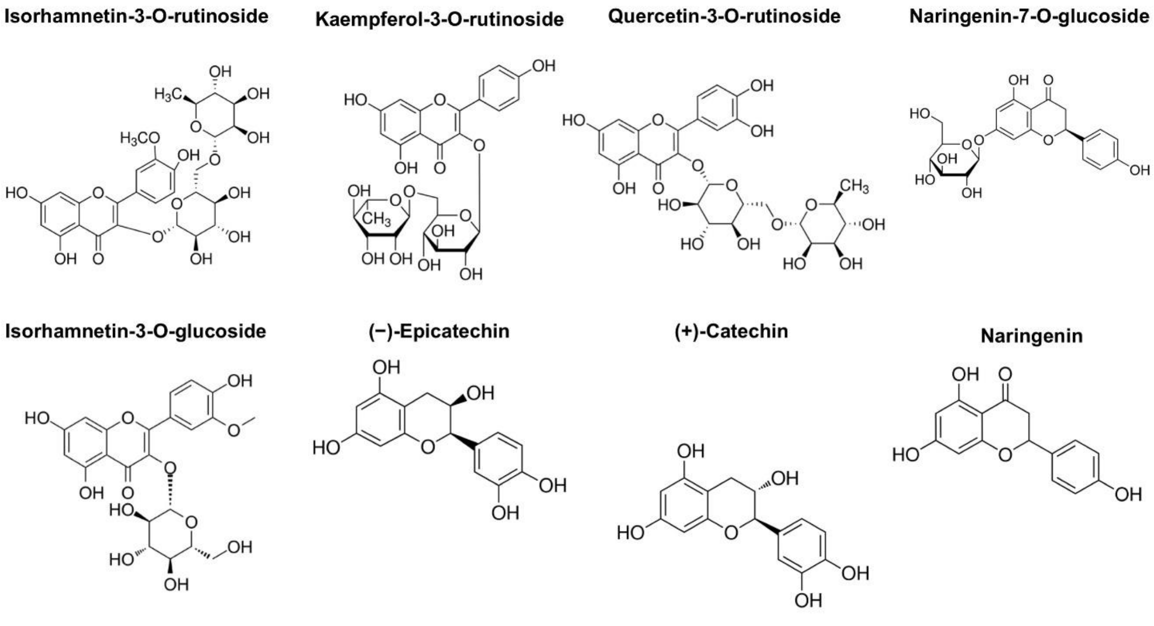

Figure S2: ATR-FTIR analysis of PCL and PCL/ASE composite films,

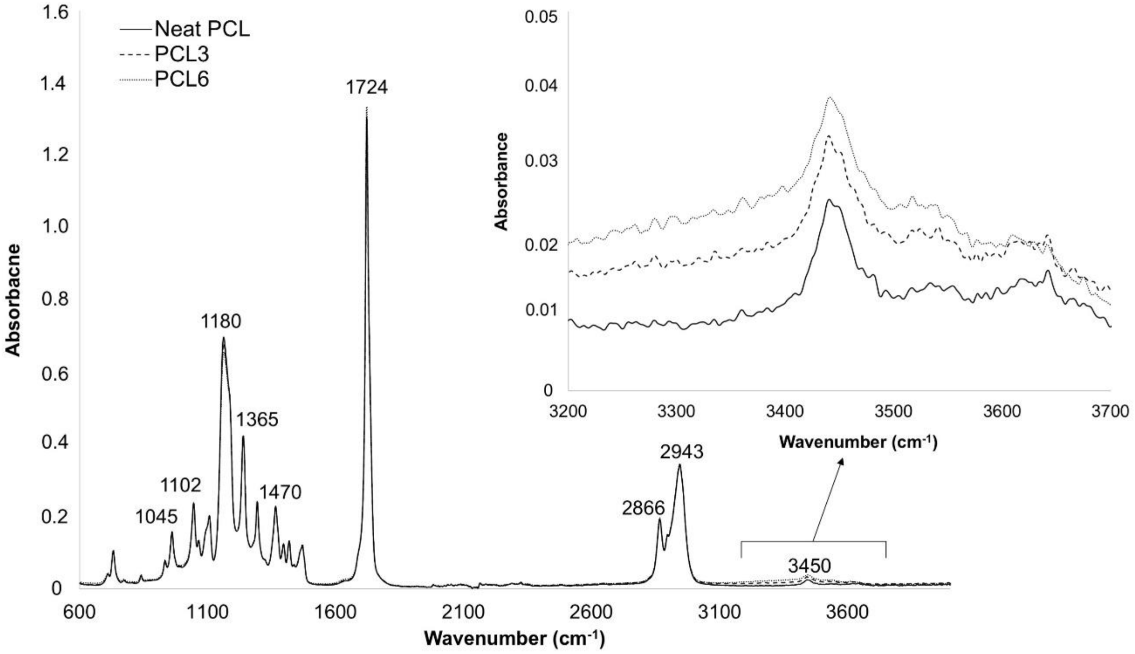

Figure S3: DTGA curve obtained for ASE,

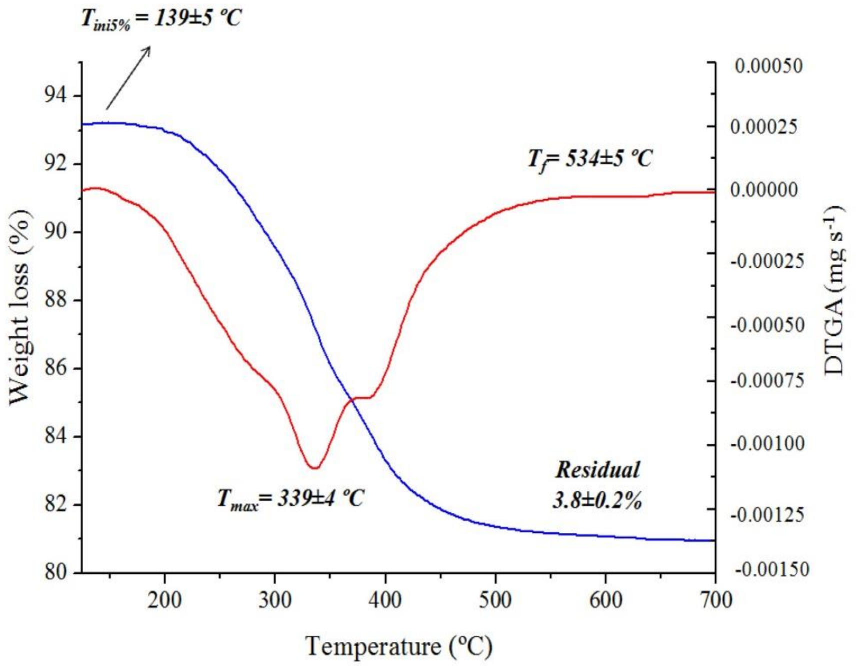

Figure S4: ATR-FTIR analysis of fried packaged almonds in PCL and PCL/ASE composite films with storage time.

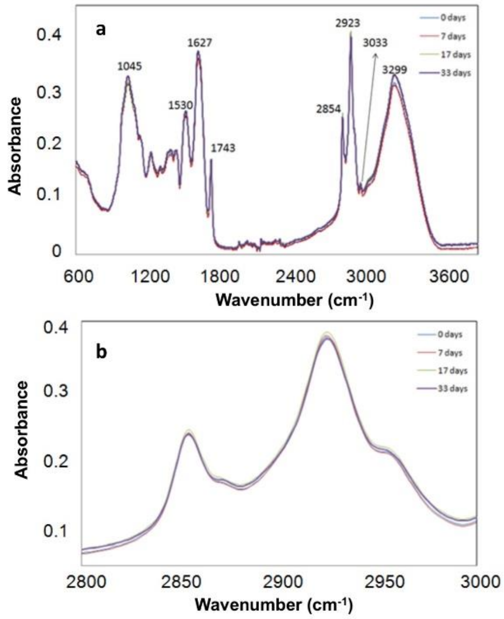

Supplement: Supplementary file 1 [file antioxidants-09-00629-s001.pdf]
